# Enhancing AAV-microdystrophin gene therapy after repeat dosing by blocking phagocytosis

**DOI:** 10.3389/fimmu.2025.1527840

**Published:** 2025-03-03

**Authors:** Rita Spathis, Deeva Robles Kuriplach, Sabrina Narvesen, Matthew Eybs, Karen Huang, Steven Torres, Madison King, Elizabeth Bagley, Pia Elustondo, Michael W. Lawlor, Kanneboyina Nagaraju, Melissa Morales

**Affiliations:** ^1^ Department of Pharmaceutical Sciences, School of Pharmacy and Pharmaceutical Sciences, Binghamton University, Binghamton, NY, United States; ^2^ AGADA Biosciences, Halifax, NS, Canada; ^3^ Department of Pathology & Laboratory Medicine, Medical College of Wisconsin and Diverge Translational Science Laboratory, Milwaukee, WI, United States

**Keywords:** AAV9, *mdx*, Duchenne muscular dystrophy, DMD, gene therapy, immune response, TLR, complement

## Abstract

**Background:**

Inefficient transduction is a major limitation in achieving therapeutic levels of AAV-delivered microdystrophin capable of improving muscle function in patients with Duchenne muscular dystrophy. Additionally, some patients experience acute complications due to activation of innate immune pathways, such as complement. We propose that inhibiting complement receptor 1/2/3 (CR 1/2/3)-mediated phagocytosis and endosomal TLR 7/8/9 signaling pathways may decrease immune and inflammatory responses while simultaneously increasing the availability of AAV virus for muscle transduction.

**Methods:**

*Mdx* mice were randomly assigned to the following three experimental conditions (n=8-9/group): Group 1, *mdx* untreated; Group 2, *mdx* + rAAV9-microdystrophin; Group 3, *mdx* + rAAV9-microdystrophin + semiweekly dosing of TLR 7/8/9 antagonist + complement receptor antibodies (combination therapy). The rAAV9-microdystrophin was administered twice to 6- and 12-week-old mice. A separate group of 6-week-old mice received a single rAAV9-microdystrophin dose and no other treatment (Group 4). We assessed several immune and inflammatory responses and dystrophin expression in the muscle.

**Results:**

Viral load was significantly increased by 77-fold in white blood cells after two rAAV9-microdystrophin doses compared to mice receiving a single dose. Repeated gene therapy resulted in a lower viral load and microdystrophin expression in muscle compared to a single rAAV dose. 63% of mice treated with two rAAV9-microdystrophin doses produced antibodies to dystrophin, which was less in mice treated with two rAAV9-microdystrophin doses and combination therapy (25%). Likewise, AAV capsid specific antibody levels were reduced in mice receiving combination therapy. Microdystrophin expression in skeletal muscle evaluated by mass spectrometry, immunofluorescence, and western blotting showed significantly higher levels in combination-treated mice compared to rAAV9-microdystrophin alone.

**Conclusions:**

Our results demonstrate that combination treatment with complement receptor 1/2/3 antibodies and a TLR 7/8/9 antagonist enhances rAAV9-microdystrophin gene therapy in *mdx* mice by partially reducing inflammatory and immune responses and increasing microdystrophin expression in skeletal muscle. Furthermore, repeated gene therapy is associated with greater uptake by white blood cells and less microdystrophin expression in the skeletal muscle. This suggests that blocking complement receptors and/or TLR 7/8/9 pathways would be a promising strategy to enhance AAV-microdystrophin therapy.

## Introduction

Gene therapy using adeno-associated virus (AAV) vectors is a promising therapeutic strategy for treating Duchenne Muscular Dystrophy (DMD), a progressive and fatal neuromuscular disorder with limited treatment options. However, there are major challenges associated with this therapy including immune responses that compromise its efficacy, safety, and long-term use potential. Clinical trials show that achieving therapeutic microdystrophin levels necessary for improved muscle function remains a significant hurdle (1). There have also been reports of acute complications involving complement pathways in some patients (2). Moreover, anti-dystrophin immune responses observed in both clinical and animal studies will likely limit the long-term usefulness of this therapeutic approach (3–5). Strategies that enhance transduction and mitigate the associated immune responses to gene therapy are therefore needed as they should lead to improved efficacy, reduced side effects, and allow for repeat dosing (2).

**Figure 1:**
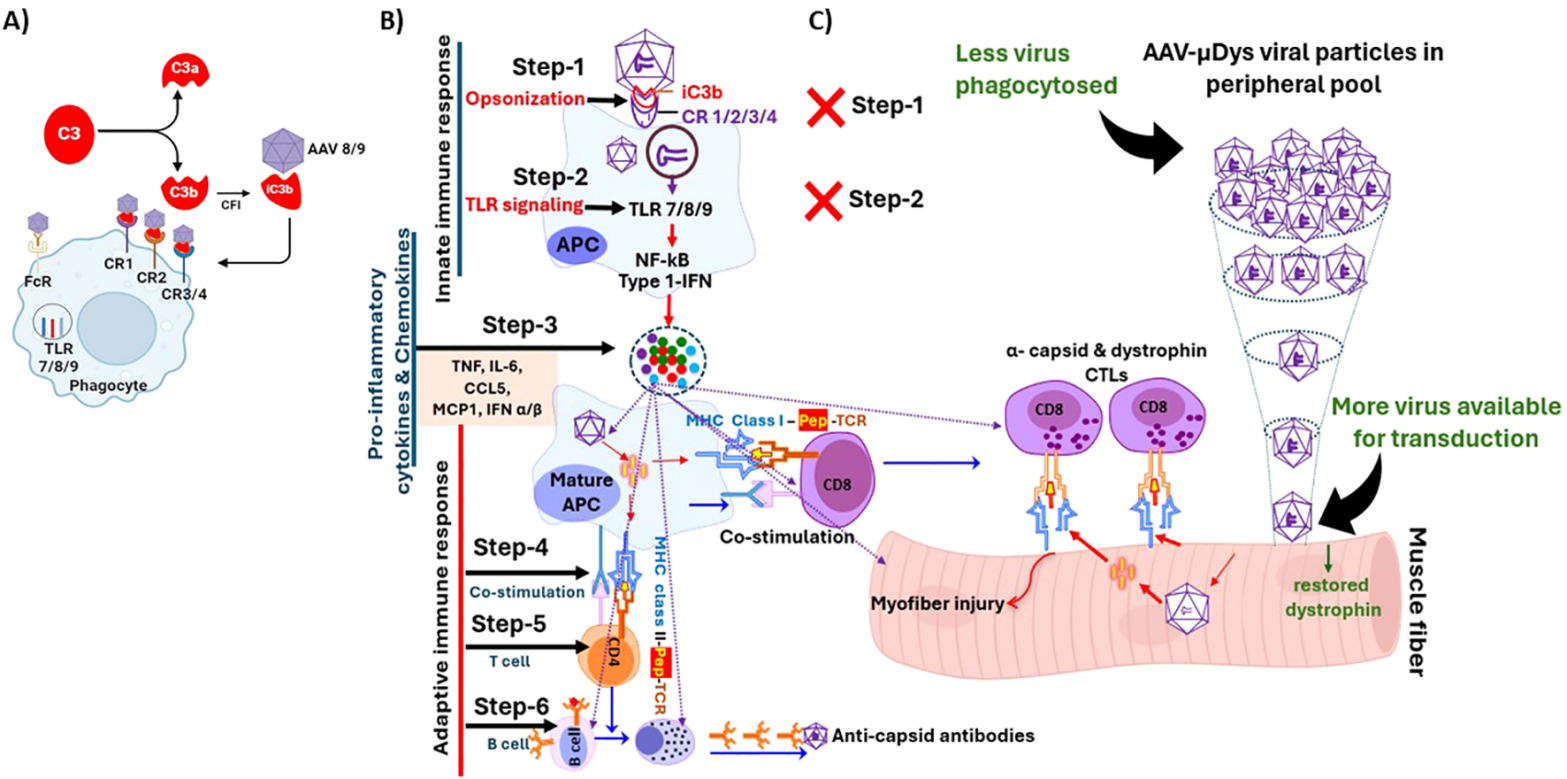
Steps involved in the initiation and perpetuation of inflammatory and immune response to rAAV virus. **(A)** iC3b serves as a key opsonin that facilitates phagocytosis through its binding to multiple complement receptors (CR), including CR 1, 2, 3, and 4. **(B)** Normal response involves phagocytosis (Step-1), endosomal TLR signaling (Step-2), pro-inflammatory cytokine production (Step-3), APC-T cell co-stimulation (Step 4), T helper and cytotoxic responses (Step-5) and cell mediated and humoral response to viral capsid and possibly expressed transgene (Step-6). **(C)** Blocking Steps 1 and 2 would abolish downstream Steps 3-6 leading to better efficacy and safety after rAAV therapy. Figure was partially created with BioRender.

We recently showed that rAAV9-microdystrophin gene therapy in *mdx* mice elicited anti-dystrophin antibodies, which were effectively eliminated by rituximab and VBP6, but not by other immunomodulatory drugs (6). We also observed that dystrophin-specific T cell responses in *mdx* mice after AAV gene therapy were not entirely suppressed by using immunomodulatory drugs such as prednisolone, rituximab and CTLA-4Ig that target effector pathways (6). These findings suggest that targeting initial inflammatory and immune responses may offer a more effective strategy to reduce immune responses to AAV gene therapy (**Figure 1**). One approach is to inhibit phagocytosis of complement-opsonized pathogens, as the complement system plays a pivotal role in the early immune response to AAV (7). Another strategy is to block the endosomal TLR 7/8/9 pathway, which is necessary for initiating pro-inflammatory and subsequent adaptive immune responses (8). We therefore investigated whether blocking these pathways could reduce immune responses triggered by rAAV-microdystrophin gene therapy and enhance muscle transduction following repeated rAAV gene therapy in *mdx* mice.

## Materials and methods

### Animal studies

All mouse experiments were performed under approved Institutional Animal Care and Use Committee (IACUC) protocols and in accordance with guidelines at Binghamton University. Five-week-old male wild type (C57BL/10ScSn/J) and *mdx* (C57BL/10ScSn-Dmd<mdx>/J) mice were purchased from The Jackson Laboratory (Bar Harbor, ME, USA). Mice were housed at Binghamton University’s animal facility under a 12 h light/dark cycle and fed a standard chow diet. All mice were acclimated to the animal facility for one week prior to any manipulation. Mice were euthanized per approved IACUC protocols and methods consistent with AVMA Guidelines on Euthanasia. Specifically, a 50% displacement rate for C02 was used, which was achieved with a 6 L/min flow rate for the mouse cage size. 

### Administration of rAAV9-microdystrophin, TLR 7/8/9 antagonist and complement receptor antibodies

**Figure 2:**
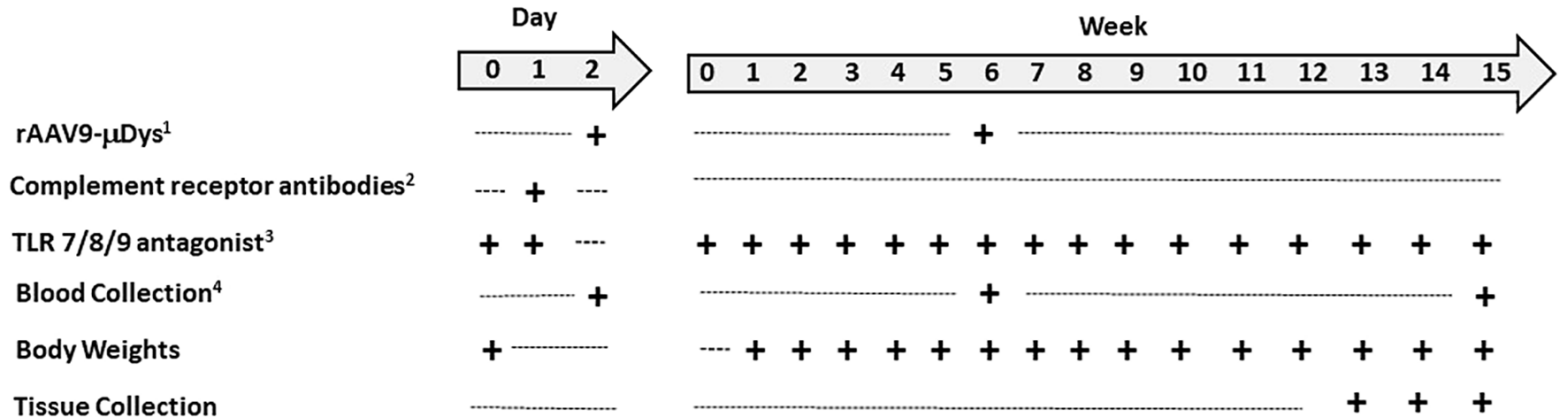
Experimental timeline for repeat rAAV-microdystrophin. Five-week-old mice arrived one week prior to the start of the experiments. ^1^AAV9-CK8-H2, 3x10^13^ vg/kg, retro-orbital injection; ^2^Includes complement receptor 1/2 and CD11b antibodies, 4 mg/kg each, in single i.p. injection; ^3^Toll-like receptor 7/8/9 antagonist, 7.5 mg/kg, i.p. injection, semiweekly after day 1 and 2 injections; ^4^Two hours post rAAV-µdys injection and at euthanasia.

Mice were randomly assigned to the following three experimental conditions (n=8-9/group): Group 1, *mdx* untreated; Group 2, *mdx* + rAAV9-microdystrophin; Group 3, *mdx* + rAAV9-microdystrophin + semiweekly dosing of TLR 7/8/9 antagonist + complement receptor antibodies (combination therapy). rAAV9-microdystrophin (Microdystrophin^ΔR4-R23/ΔCT^) (9, 10) was provided as a single batch by Solid Biosciences and administered twice to 6- and 12-week-old mice at 3x10^13^ vg/kg by retro-orbital injection. One mouse from Group 2 was removed from all analyses due to an unsuccessful rAAV9-microdystrophin injection. The TLR 7/8/9 antagonist (ChemGenes, Wilmington, MA, USA) was administered at 7.5 mg/kg intraperitoneally (i.p.) on the two consecutive days prior to the first rAAV9-microdystrophin injection, and then semiweekly until euthanasia. Complement receptor 1/2 (Clone 7G6, Absolute Antibody, UK) + CD11b (complement receptor 3) antibody (Clone M1/70, BioCell, Lebanon, NH, USA) were administered at 4 mg/kg each in a single i.p. injection on the day prior to the first rAAV9-microdystrophin dose. The dose and frequency of these drugs were based on the literature, including our use of TLR 7/8/9 antagonists in *mdx* mice (11–13). A separate group of 6-week-old mice received a single rAAV9-microdystrophin dose and no other treatment (Group 4). The experimental timeline for repeated rAAV9-microdystrophin dosing is shown in **Figure 2**.

### Quantification of viral load in the blood and muscle

DNA was purified from white blood cells collected 2 hours after the rAAV9-microdystrophin injection or from the tibialis anterior muscle collected at euthanasia. Total DNA was subjected to Taqman qPCR, and the number of viral genomes was determined by generating a standard curve using titrated AAV9-CK-H2 plasmid (kindly provided by Jessica Boehler).

### Anti-dystrophin and anti-capsid antibody detection

#### Anti-dystrophin antibodies

Total protein was extracted from dystrophin sufficient skeletal muscle (kindly provided by Eric Hoffman) and 50 μg was resolved through NuPage™ Tris-Acetate 3-8% gels (Thermo Fisher Scientific, USA) followed by electro-blotting overnight onto nitrocellulose membranes. Membranes were stained with Ponceau S (Thermo Fisher Scientific) to assess the equality of transfer and subsequently cut into individual strips. Each strip was incubated overnight at 4°C with individual mouse sera (1:100). The presence of dystrophin was confirmed by incubating one strip with the anti-dystrophin antibody GTX15277 (GeneTex, Inc.). The blots were washed and incubated with HRP-conjugated secondary antibody and the chemiluminescent signal detected with ECL reagent (Amersham BioSciences).

#### Anti-capsid antibodies

Sera (1:20,000) from terminal cardiac puncture were applied in duplicate to 96-well Meso Scale Discovery (MSD) plates coated with 5 x10^8^ viral particles/well of AAV9 empty capsid (Charles River). MSD Sulfo-TAG labeled goat-anti mouse secondary antibody was added to each well and incubated for 1 hour. Bound signal (RFU) was detected on the MESO QuickPlex plate reader.

### Dystrophin expression

#### Mass spectrometry

For quantification of microdystrophin via mass spectrometry, equal protein aliquots of quadriceps muscle extract were spiked with isotopically labeled dystrophin peptide VGNILQLGSK (VGN) and samples digested with trypsin. The intensity of the heavy to light peptide signal was used to calculate the femtomole (fmol) of microdystrophin in each sample.

#### Immunofluorescence

Isopentane-frozen quadriceps and tibialis anterior muscle was sectioned to 8-micron thickness and stained for dystrophin/microdystrophin (DysB, Leica Biosystems). Stained tissues were imaged on an Olympus VS120 slide scanner microscope and evaluation of percent dystrophin or microdystrophin positive fibers was performed in a blinded fashion using Qupath analysis software.

### Western blot

10 µg quadriceps muscle extracts were resolved through NuPage™ 4-12% Bis-Tris gels (Thermo Fisher Scientific, USA) followed by electro-blotting overnight onto nitrocellulose membranes. Membranes were stained with Ponceau S (Thermo Fisher Scientific) to assess the equality of transfer and then incubated overnight at 4°C with either dystrophin specific DysB monoclonal antibody (1:100; Leica Biosystems) or GAPDH antibody (1:2000; Cell Signaling Technology). The blots were incubated with HRP-linked secondary antibodies and signal detected with ECL reagent (Amersham Biosciences).

### Histological evaluation of inflammation

H&E: Slides were prepared and stained by Histoserv Inc. (Gaithersburg, MD). Digital images from each animal were acquired in 20X using an Olympus VS120 slide scanner microscope. One entire transverse quadricep muscle section was analyzed per animal in a blinded fashion. Inflammation foci (group of ≥10 inflammatory cell nuclei) were counted and normalized to the entire tissue section (mm^2^) (14). Immunofluorescence: Quadriceps muscles were cryosectioned and co-stained overnight at 4°C with the monoclonal antibodies, F4/80 (1:100; D2S9R; Cell Signaling Technology) and DysB (1:25; Leica Biosystems) targeting macrophages and dystrophin-respectively. On the following day, secondary antibodies for F4/80 and DysB, Alexa Fluor 568 goat anti rabbit IgG and Alexa Fluor 488 goat anti mouse IgG1, respectively were applied (both at 1:1200) with DAPI (2µg/ml) for 1h at room temperature. Slides were then mounted with ProLong™ Gold anti-fade reagent (ThermoFisher) and digital images were acquired in 20X using an Olympus VS120 slide scanner microscope.

### Liver enzyme levels

Alanine aminotransferase (ALT) and aspartate aminotransferase (AST) levels were assayed in duplicate from terminal cardiac sera using commercially available ELISA kits (Abcam) according to manufacturer’s instructions.

### Creatine Kinase

Creatine kinase (CK) levels were assayed from terminal cardiac sera using a commercially available kit (SEKISUI Diagnostics, LLC) according to manufacturer’s instructions.

### Statistical analysis

Data with two groups were analyzed via Student’s *t* tests. Data with three or more groups were analyzed via one-way ANOVA, except for repeated measures data (e.g., body weight gain), which was analyzed by a mixed-effects ANOVA. Non-parametric tests were used if the data were not normally distributed. Outliers by individual samples and outcome measures were removed prior to data analysis. Data are presented as mean ± standard error of the mean (M ± SEM) and significance was set as a *p*-value less than 0.05. All data were analyzed using Prism software (GraphPad, La Jolla, CA).

## Results

### Body and tissue weight

**Figure 3:**
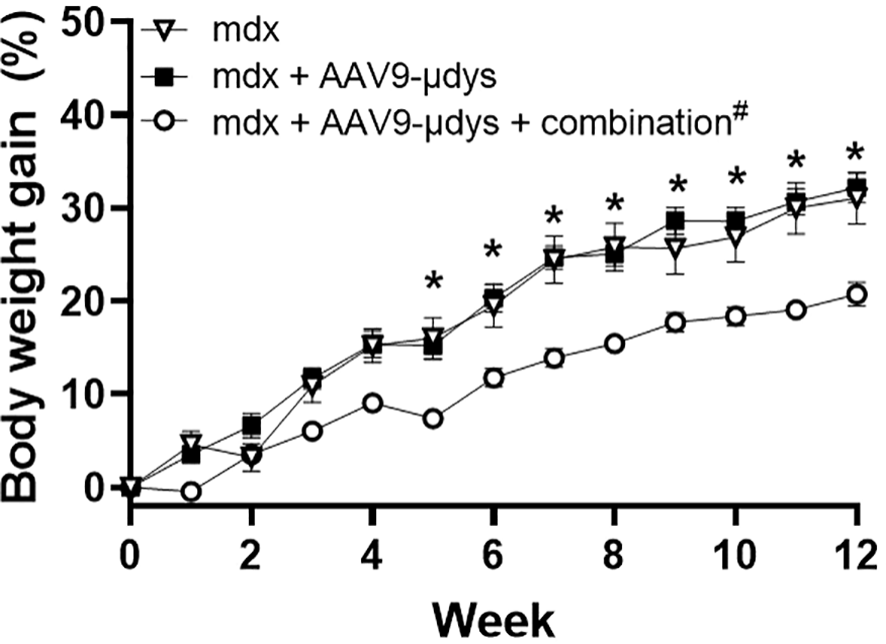
Body weight gain during treatment. Body weight was recorded weekly during the experiment. AAV9-µDys, rAAV9-microdystrophin; ^#^combination includes complement receptor 1/2 and CD11b antibodies and Toll-like receptor 7/8/9 antagonist. Data are presented M ± SEM. *p<0.05 for *mdx* + AAV9-µDys + combination compared to untreated *mdx* and *mdx* + AAV9-µDys.

Body weight data revealed significant main effects of week (p<0.0001) and treatment (p<0.001), and an interaction between week and treatment (p<0.0001). All groups significantly gained weight from week 1 to week 12 (**Figure 3**). By week 5 and throughout the remainder of the study, *mdx* mice injected with rAAV9-microdystrophin plus combination therapy (CR 1/2/3 + TLR 7/8/9) weighed significantly less than untreated *mdx* and *mdx* + rAAV9-microdystrophin (p<0.005) (**Figure 3**). Tissue weights were normalized to body weight at euthanasia. Overall, skeletal muscle weights were lower in rAAV9-microdystrophin plus combination therapy mice (**Supplementary Table 1**).

### AAV uptake in the blood and muscle

**Figure 4:**
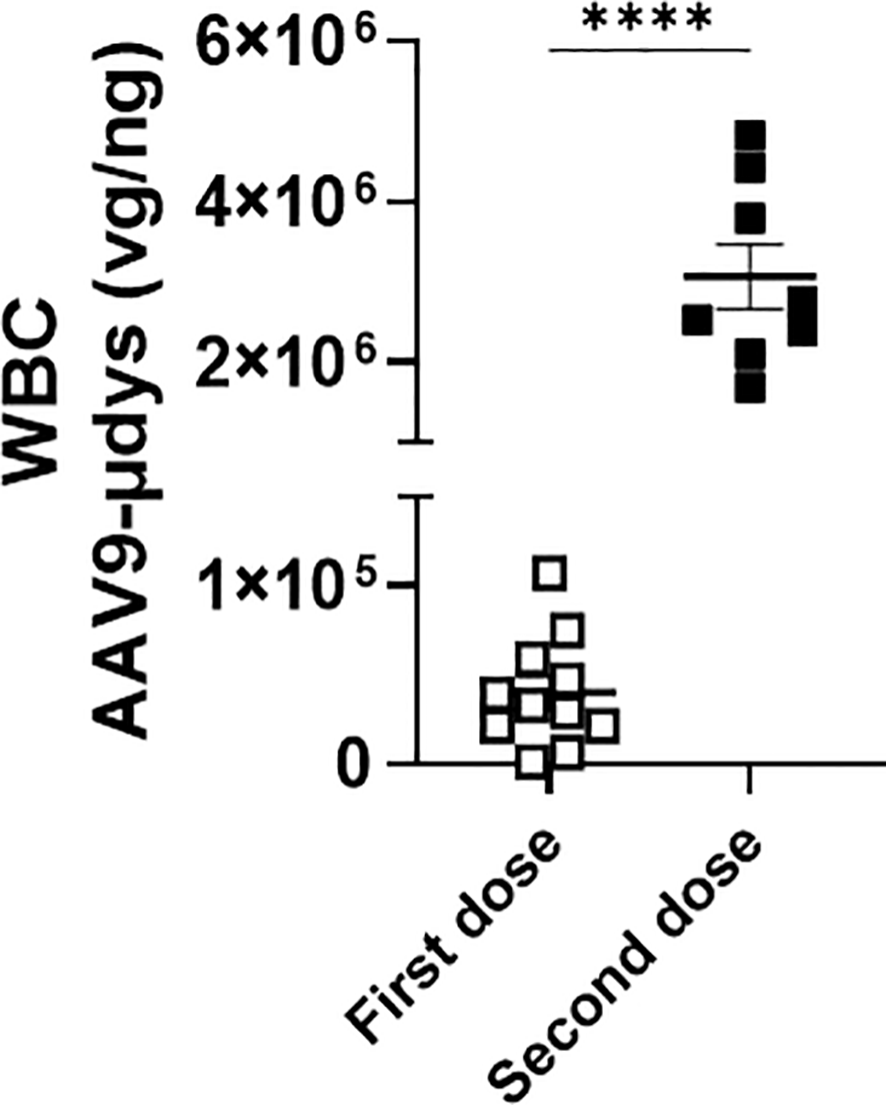
Repeat dosing significantly increases AAV uptake by white blood cells. DNA was purified from blood collected 2 hours after the first (cohort 1) or second (cohort 2) rAAV9-microdystrophin (week 1 and 6) injection. WBC, white blood cells. Data are presented M ± SEM. ****p<0.0001.

We first compared viral load in white blood cells from a cohort of mice injected once with rAAV9-microdystrophin (first dose) and another cohort injected twice with rAAV9-microdystrophin (second dose), separated by six weeks. AAV uptake was significantly increased 77-fold (p<0.0001) after two rAAV9-microdystrophin doses compared to a single dose (**Figure 4**). AAV9 viral load in the tibialis anterior (TA) muscle was significantly lower in mice receiving two rAAV9-microdystrophin doses compared to mice receiving one dose (**Supplementary Figure 1**). We also examined the effect of combination therapy on viral load in the skeletal muscle from the cohort of mice receiving two rAAV9-microdystrophin doses. Combination therapy did not significantly increase AAV viral load in the TA muscle (2.0-fold increase) compared to the group receiving only rAAV9-microdystrophin (data not shown).

### Anti-dystrophin antibodies

**Figure 5:**
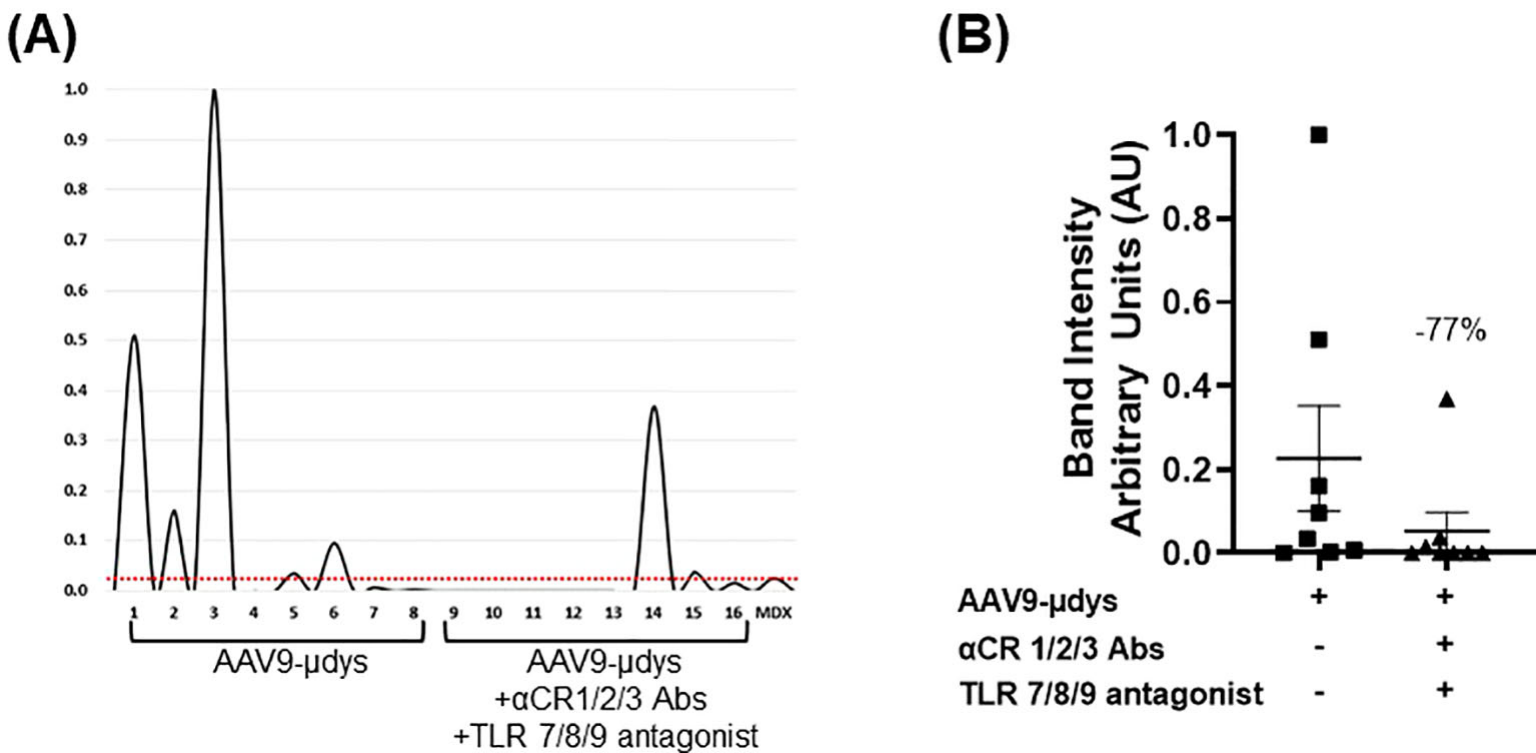
Combination treatment reduces the frequency and presence of dystrophin-reactive antibodies. Protein extracted from dystrophin sufficient skeletal muscle was incubated overnight with terminal mouse serum (1:100 dilution). The presence of dystrophin was confirmed by incubating one strip with the commercially available antibody GTX15277 (GeneTex, Inc.). Bands were analyzed using AzureSpot Pro software v1.4. Horizontal red line indicates threshold limit based on *mdx* intensity. AAV9-µDys, rAAV9-microdystrophin; αCR 1/2/3, includes complement receptor 1/2 and CD11b antibodies; TLR 7/8/9 antagonist, Toll-like receptor 7/8/9 antagonist. Data are presented M ± SEM. * p<0.05.

Anti-dystrophin antibody response was evaluated by western blot. Antibodies were detected in 5 out of 8 (62.5%) mice treated with rAAV9-microdystrophin, compared to 2 out of 8 (25%) in mice injected with rAAV9-microdystrophin and combination therapy (**Table 1**). Quantification of band intensity showed a non-significant decrease (p=0.07) in mice receiving combination therapy. Densitometry analysis of western blot anti-dystrophin antibody responses and analysis of band intensity are shown in **Figures 5A, B**, respectively.

**Table 1:** Frequency of mice expressing anti-dystrophin antibodies.

Treatment Group	Tested	Positive	% Positive
AAV9-µdys	8	5	62.5
AAV9-µdys + αCR1/2/3 Abs +TLR 7/8/9 antagonist	8	2	25.0

### Anti-capsid antibodies

Anti-capsid antibodies were evaluated by MSD. We first compared capsid-specific antibodies from a cohort of mice injected once with rAAV9-microdystrophin (first dose) and another cohort injected twice (second dose), separated by six weeks. Mice injected twice with rAAV9-microdystrophin had significantly higher levels of anti-capsid antibodies compared with mice injected once with a single dose (**Supplementary Figure 2**). The average relative fluorescent signal following rAAV9-microdystrophin and combination treatment was reduced by 26% (19669 ± 3526) compared to rAAV9-microdystrophin treated mice (26468 ± 3644), but this did not reach statistical significance (**Supplementary Figure 3**).

### Dystrophin expression

**Figure 6:**
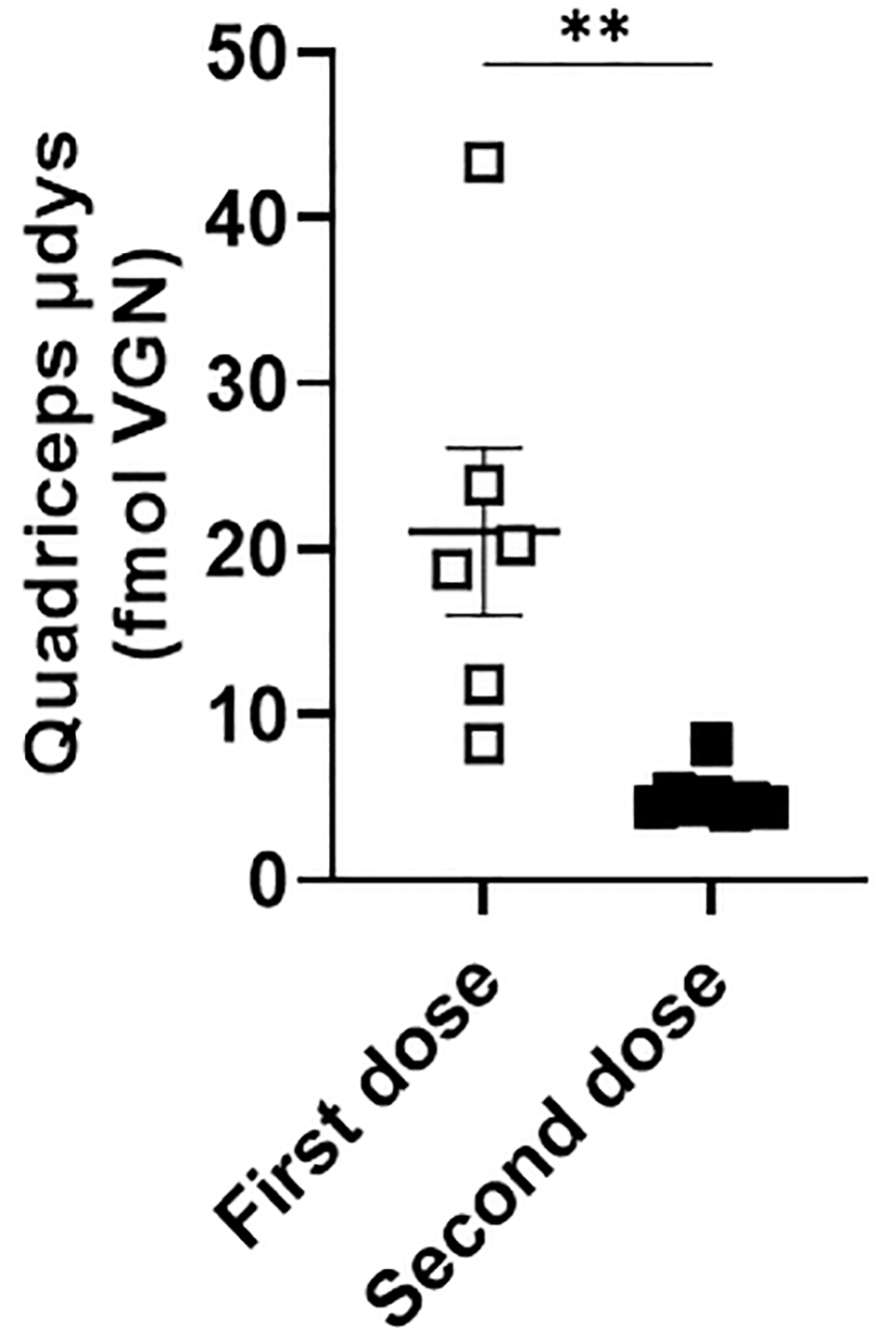
Microdystrophin expression levels in the quadriceps is lower in mice receiving two rAAV doses. Dystrophin from the quadriceps measured by mass spectrometry is presented as average fmol dystrophin ± SEM. rAAV9-microdystrophin was injected into a cohort of mice receiving one dose and another cohort of mice receiving two doses. Data are presented M ± SEM. ** p<0.01.

**Figure 7:**
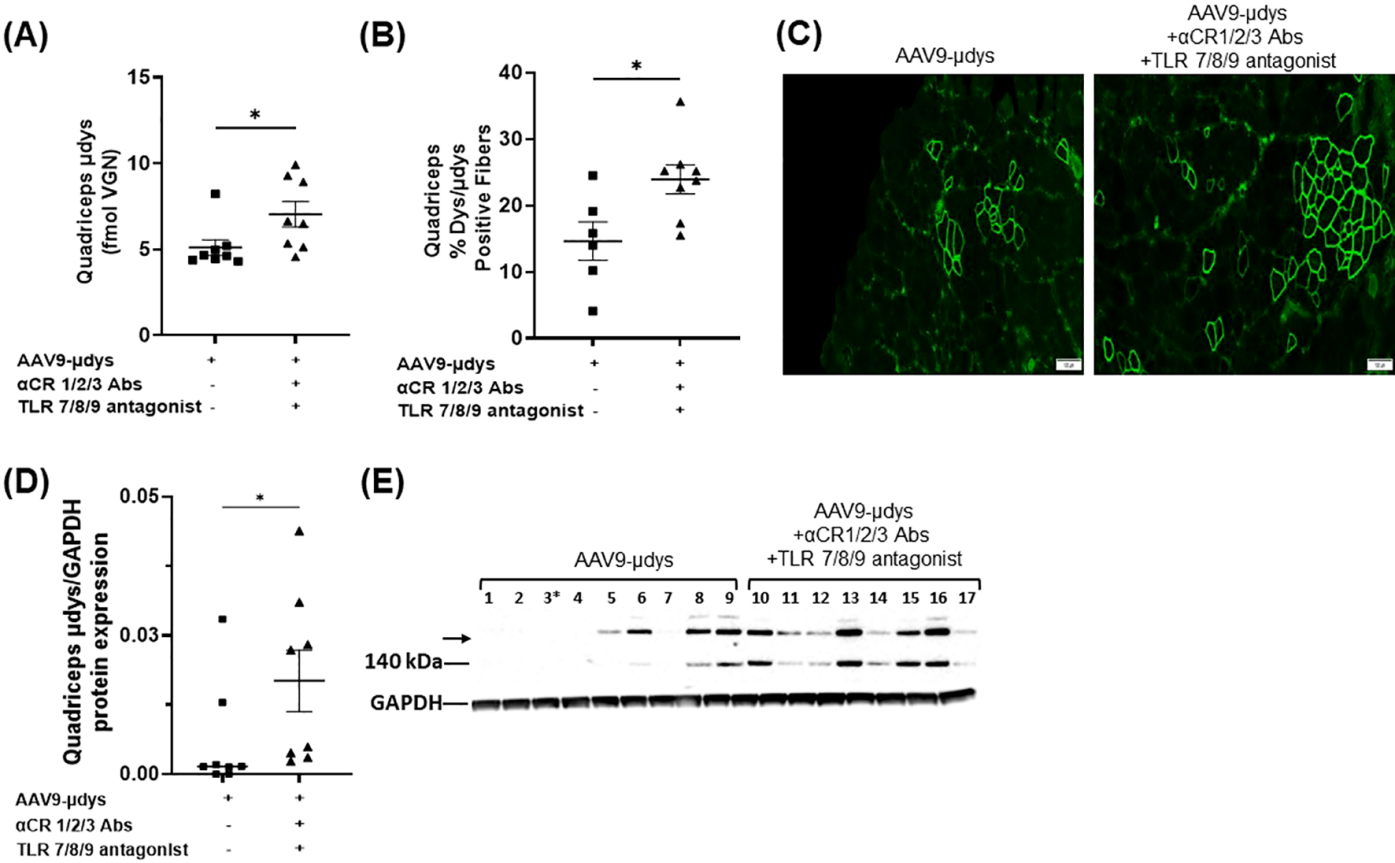
Microdystrophin expression by mass spectrometry, immunofluorescence, and western blot is increased in mice receiving combination therapy. Dystrophin from the quadriceps measured by mass spectrometry is presented as average fmol dystrophin ± SEM. For immunofluorescence, percentage of dystrophin/µdystrophin positive fibers in quadriceps. For microdystrophin protein expression, band intensity was analyzed using AzureSpot Pro software v1.4 and the ratio of μdys/GAPDH was used for quantification. Western blot shows all samples, including sample 3*, which was excluded from all analyses due to an unsuccessful injection. The μdys band is shown at 140 kDa and was used for μdys/GAPDH ratio calculation. The upper band migrating slightly above 198 kDa (→) is dimerized μdys and was not included in the statistical analysis. AAV9-µDys, rAAV9-microdystrophin; αCR 1/2/3, includes complement receptor 1/2 and CD11b antibodies; TLR 7/8/9 antagonist, Toll-like receptor 7/8/9 antagonist. Data are presented M ± SEM. *p<0.05.

Dystrophin expression was measured using mass spectrometry (MS), immunofluorescence (IF), and western blot. We first compared microdystrophin expression by MS in the skeletal muscle from a cohort of mice injected once with rAAV9-microdystrophin (first dose) and another cohort injected twice with rAAV9-microdystrophin (second dose), separated by six weeks. Microdystrophin expression levels in the quadriceps muscle were significantly higher in the cohort of mice receiving one rAAV9-microdystrophin dose compared to mice receiving two rAAV9-microdystrophin doses (**Figure 6**). We then examined the effects of combination therapy on microdystrophin expression using several methods. The MS analysis showed that mice in the combination treatment group had significantly higher levels of microdystrophin expression in the quadriceps muscle than mice injected with rAAV9-microdystrophin alone (p<0.05) (**Figure 7A**). The IF analysis of percentage of microdystrophin positive fibers was significantly greater in the combination treated mice compared to rAAV9-microdystrophin only treated mice (p<0.05) (**Figure 7B**). Representative images showing IF dystrophin expression in the quadriceps are shown in **Figure 7C**. We also evaluated IF microdystrophin expression in the TA muscle and found a similar pattern, with a 2-fold increase (p=0.09) of microdystrophin positive fibers in mice receiving combination treatment compared to rAAV9-microdystrophin alone (data not shown). Microdystrophin protein levels in the quadriceps was significantly increased in mice receiving combination therapy compared to mice receiving rAAV9-microdystrophin alone (**Figures 7D, E**).

### Muscle histology

**Figure 8:**
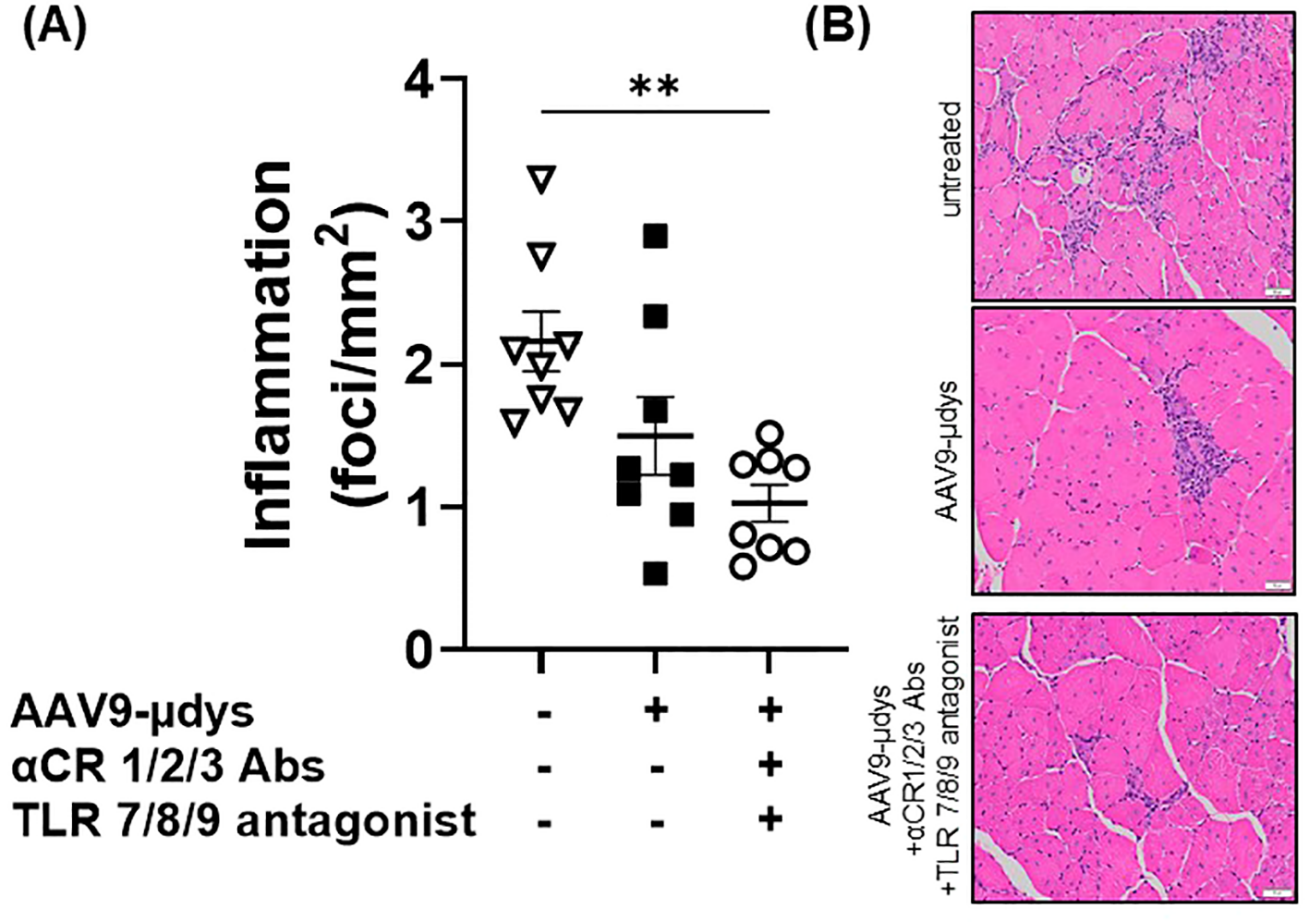
Inflammatory foci in H&E-stained quadriceps are lower after combination therapy. Inflammatory foci (group of ≥10 inflammatory cell nuclei) were counted and normalized to the entire tissue section (mm^2^). AAV9-µDys, rAAV9-microdystrophin; αCR 1/2/3, includes complement receptor 1/2 and CD11b antibodies; TLR 7/8/9 antagonist, Toll-like receptor 7/8/9 antagonist. **p<0.01.

**Figure 9:**
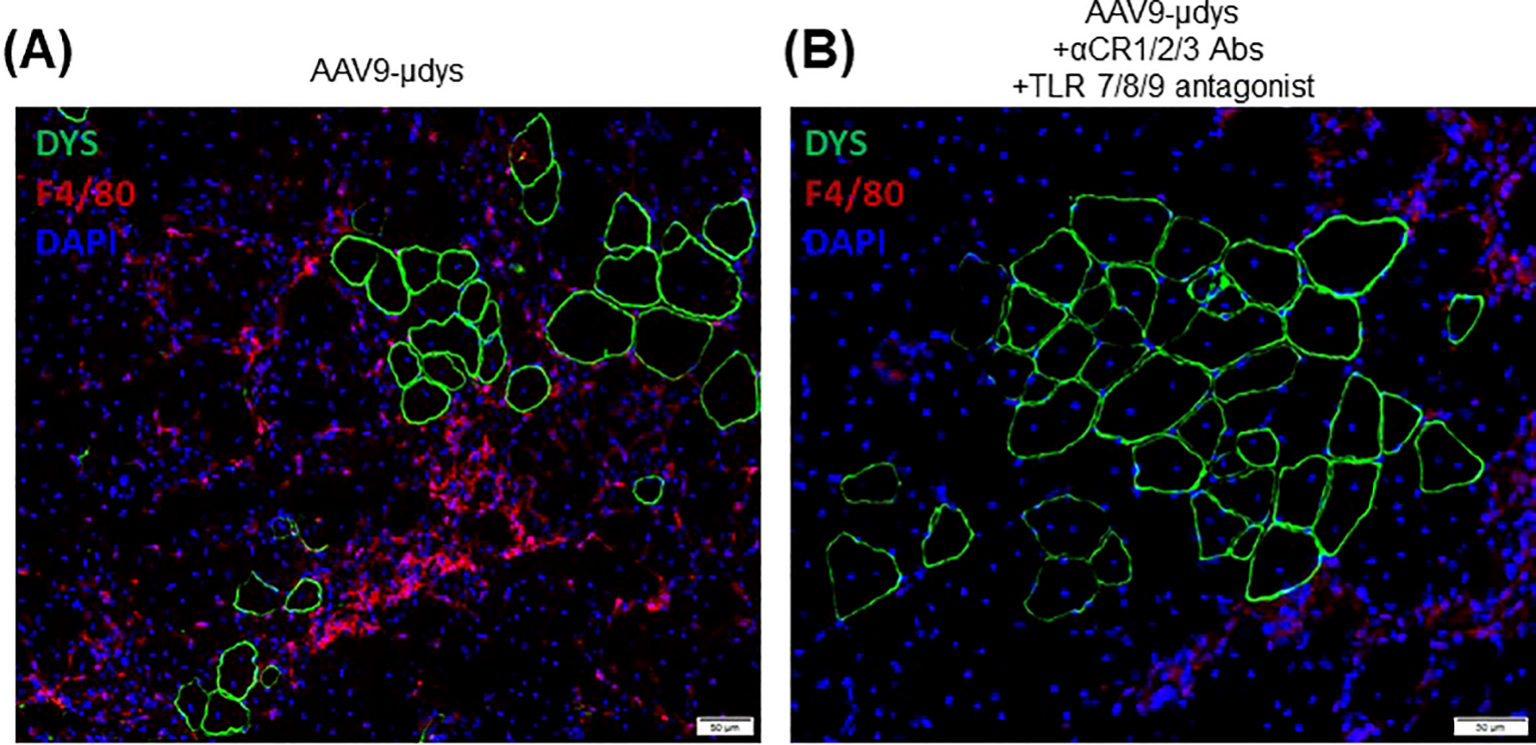
Macrophage infiltration and dystrophin staining in the quadriceps muscle. Immunofluorescence co-staining of dystrophin (green) and macrophages (F4/80, red), with DAPI staining of nuclei shown in blue. Representative images of a mouse treated with rAA9-μdys alone **(A)** and one treated with combination therapy **(B)**.

Mice in the rAAV9-microdystrophin group showed a non-significant reduction (p=0.09) in inflammatory foci per mm^2^ compared to untreated *mdx* mice (**Figure 8A**). Mice in the rAAV9-microdystrophin and combination treatment group had significantly less inflammation compared to untreated *mdx* mice (p<0.01). Representative images showing inflammation on H&E-stained sections are shown in **Figure 8B**. Macrophage infiltration was observed in the quadriceps muscle in areas without dystrophin positive staining whereas less macrophages were present in areas with dystrophin positive staining (**Figures 9A, B**).

### Serum creatine kinase

Analysis of serum CK at euthanasia revealed no significant differences between groups (**Supplementary Figure 4**).

### Liver enzymes

There were no significant differences observed in ALT or AST levels between groups (**Supplementary Figure 5**).

## Discussion

In the current study, we investigated the therapeutic potential of blocking early steps in the host immune response to AAV gene therapy to decrease immune activation and increase muscle transduction. Specifically, we inhibited complement-mediated phagocytosis and endosomal TLR signaling pathways. Our results demonstrate that treatment with complement receptor 1/2/3 antibodies and a TLR 7/8/9 antagonist enhances rAAV9-microdystrophin gene therapy in *mdx* mice by partially reducing inflammatory and immune responses and increasing microdystrophin expression in skeletal muscle.

We first investigated differences in AAV uptake in mice treated with one compared to two doses of rAAV9-microdystrophin. We found that there was a significant increase in AAV uptake by phagocytes in mice who received a second rAAV9-microdystophin dose compared to mice receiving only a single dose. We postulate that after the first dose, the majority of AAV uptake is mediated by complement receptors, whereas following the second dose, it is Fcγ receptors that contribute to the increased uptake. This is supported by a recent study demonstrating that after a second rAAV9-microdystrophin dose, there was a significant increase in anti-AAV IgGs along with activated monocytes expressing both Fcγ and complement receptors (15). Therefore, increased AAV uptake by phagocytes after two AAV doses should result in a lower viral load in the skeletal muscle compared to mice receiving a single dose. This is indeed what we observed (**Supplementary Figure 1**). Additionally, when we examined microdystrophin expression in the skeletal muscle of these mice, lower levels were observed in the quadriceps muscle from mice receiving two AAV doses compared to one (**Figure 6**). Altogether, these data suggest that after two AAV doses, most of the virus is engulfed by phagocytes, leaving less available for muscle transduction. This may also explain the variability in microdystrophin expression observed between the two rAAV-microdystrophin injected cohorts of mice.

AAV uptake occurs through various endocytic routes, including receptor and non-receptor mediated phagocytosis (7, 16–18). After gene therapy administration, a substantial portion of the AAV virus is quickly cleared from circulation by phagocytosis thus limiting availability of the virus to enter target cells. Blocking this phagocytic process has been shown to improve the efficacy of AAV mediated gene therapy *in vivo* (19). One critical phagocytic mechanism is mediated by complement receptors via opsonization. Although there are three complement pathways which are distinct in their activation, all lead to the generation of C3 convertase, which cleaves C3 to form C3a and C3b, with the latter facilitating phagocytosis of opsonized targets (20). Opsonization consists of complement component iC3b binding to viral particles and complement receptors 1/2 on phagocytic cells (7, 21). The complement product, iC3b is a major opsonin due to its ability to bind multiple complement receptors to facilitate phagocytosis (22) (**Figure 1A**). Taking these interactions into account, we used complement receptor antibodies to CR 1/2/3 to inhibit phagocytosis (**Figure 1B**, Step 1). However, blocking complement receptors does not fully inhibit all phagocytic processes, and activation of TLR 7/8/9 still occurs, leading to stimulation of NF-KB, type 1 interferon, and pro-inflammatory cytokines (**Figure 1B**). Therefore, to further inhibit immune and inflammatory pathways, we also blocked the next step of the immune response to AAV with a TLR 7/8/9 antagonist (**Figure 1B**, Step 2) which we previously showed to reduce muscle inflammation in *mdx* mice (11).

Based on this, we expected that treating mice with complement receptor 1/2/3 antibodies and a TLR 7/8/9 antagonist would improve muscle transduction and increase microdystrophin expression in the skeletal muscle after two rAAV doses. We observed that mice receiving two rAAV9-microdystrophin doses and combination therapy showed a 37% increase in rAAV-microdystrophin viral genomes in the tibialis anterior, although this did not reach statistical significance (data not shown). This suggests that blocking complement-receptor mediated phagocytosis and endosomal TLR activation partially enhances muscle transduction and could be a promising therapeutic approach to increasing viral particles that effectively target muscle tissue. The FDA’s recent accelerated approval of Elevidys, the first approved AAV gene therapy for DMD patients was based on the surrogate endpoint of increased microdystrophin expression (23). In the current study, microdystrophin expression in the quadriceps muscle of *mdx* mice by three methods, including mass spectrometry, immunofluorescence, and western blot was significantly higher in mice treated with AAV9-microdystrophin and combination therapy compared to mice treated with rAAV9-microdystrophin alone. Although we did see significant improvement, geographical variability in microdystrophin expression exists, and the improvement was partial; therefore, complete transient blockade has the potential to further increase dystrophin expression. We previously showed that blocking downstream immune responses with several immunomodulatory drugs was not sufficient to enhance microdystrophin expression after a single rAAV9-microdystrophin dose (6). Together, these results suggest that we can increase microdystrophin expression in the skeletal muscle by blocking initial innate immune responses by inhibiting macrophage opsonization and TLR activation. There are currently several clinical trials underway using C3 inhibitors (e.g., AMY-101 and APL-2) and TLR 7/8/9 antagonists (e.g., IMO-8400) for various inflammatory and autoimmune diseases (24–26), indicating that these drugs may be readily incorporated into DMD gene therapy clinical trials.

Dystrophin replacement by gene therapy triggers an anti-dystrophin immune response in DMD patients and animal models that likely limits the long-term efficacy of this treatment (3–5, 27). Our laboratory previously observed an increased frequency of *mdx* mice expressing anti-dystrophin antibodies following rAAV9-microdystrophin treatment, which was reduced after treatment with rituximab, VBP6, and prednisolone (6). Similarly, VBP6 and prednisolone effectively mitigated dystrophin-specific T-cell responses (6). Findings from the Elevidys trial also reported minimal T-cell responses against microdystrophin, which was likely attributed to corticosteroid treatment before and after gene therapy (28). Notably, there were large increases in T-cell responses directed against the viral capsid (28). Our current study showed that blocking complement receptor antibodies and antagonizing TLR 7/8/9 pathways reduced the number of mice developing both anti-dystrophin and anti-capsid antibodies compared to mice treated with rAAV9-microdystrophin alone. Importantly, we observed significantly higher anti-capsid antibody levels in mice injected twice with rAAV9-microdystrophin compared to mice injected once (**Supplementary Figure 2**), indicating an elevated immune response to the capsid after repeat dosing. Furthermore, we observed decreased inflammation in the quadriceps by histological evaluation in mice who received combination therapy compared to untreated *mdx* mice. This suggests that dystrophin restoration lessens muscle inflammation. Although we observed a non-significant reduction in muscle inflammation in rAAV9-microdystrophin treated mice, this lack of significance could be due to duration of treatment and dose. Furthermore, we observed that macrophage infiltration was less apparent in areas of dystrophin restoration (**Figure 9**), but are present in areas without dystrophin correction, supporting our previous findings after exon-skipping (29). Overall, we demonstrated improvements in inflammatory and immune responses following this combined treatment approach in *mdx* mice receiving two doses of rAAV9-microdystrophin.

Analysis of body weight gain revealed that mice treated with complement receptor antibodies and the TLR 7/8/9 antagonist weighed significantly less beginning at week 5 compared to untreated *mdx* mice and those treated with rAAV9-microdystrophin alone. This corresponded with reduced weights of skeletal muscles (triceps, quadriceps, and tibialis anterior) in the combination therapy group, although significant reductions were observed only in the triceps. Typically, body and skeletal muscle weights in *mdx* mice are higher than those in wild-type controls (6, 30, 31), and previous reports from our lab have demonstrated that various immunomodulatory treatments lead to decreased body weight compared to untreated *mdx* mice (6). Similar trends were observed in the current study with combination therapy, suggesting that the combination treatment with complement receptor antibodies and TLR 7/8/9 antagonist restored muscle and body weights to normal B10 levels (6).

Some limitations of the current study include small sample size, single viral dose (3x10^13^ vg/kg) and a single timepoint for measurements. While our results are promising as they indicate improvement in several AAV-gene therapy responses, it is possible that we may have seen a greater improvement had we examined multiple timepoints or various doses. Additionally, the lack of statistical significance in some analyses may be due to our small sample size (n=8 per group). It is possible that increasing group sizes would have given us enough power to demonstrate further improvement. Again, despite these limitations, our results indicate that blocking complement receptors and TLR 7/8/9 pathways partially enhances gene therapy in *mdx* mice.

In the current study we showed that blocking complement receptor-mediated phagocytosis and endosomal TLR pathways partially enhances muscle transduction and microdystrophin expression while reducing the anti-capsid and anti-dystrophin immune responses. Although these data are encouraging, the increased efficacy of gene therapy observed by blocking these mechanisms was limited given that we blocked only complement-mediated phagocytosis while other endocytosis pathways of AAV uptake exist. This suggests that pharmacological approaches blocking phagocytic mechanisms more broadly may produce a greater effect in reducing inflammatory and immune activation while simultaneously increasing the availability of AAV virus for muscle transduction.

## Data Availability

The original contributions presented in the study are included in the article/**Supplementary Material**. Further inquiries can be directed to the corresponding author.
