# Corrigendum: Enhancing AAV-microdystrophin gene therapy after repeat dosing by blocking phagocytosis

**DOI:** 10.3389/fimmu.2025.1593193

**Published:** 2025-03-21

**Authors:** Rita Spathis, Deeva Robles Kuriplach, Sabrina Narvesen, Matthew Eybs, Karen Huang, Steven Torres, Madison King, Elizabeth Bagley, Pia Elustondo, Michael W. Lawlor, Kanneboyina Nagaraju, Melissa Morales

**Affiliations:** ^1^ Department of Pharmaceutical Sciences, School of Pharmacy and Pharmaceutical Sciences, Binghamton University, Binghamton, NY, United States; ^2^ AGADA Biosciences, Halifax, NS, Canada; ^3^ Department of Pathology & Laboratory Medicine, Medical College of Wisconsin and Diverge Translational Science Laboratory, Milwaukee, WI, United States

**Keywords:** AAV9, *mdx*, Duchenne muscular dystrophy, DMD, gene therapy, immune response, TLR, complement

In the published article, there was an error in the **Funding** statement. The funder “Duchenne UK” was erroneously excluded. The correct **Funding** statement appears below.

“The author(s) declare that financial support was received for the research, authorship, and/or publication of this article. The authors would like to thank the Parent Project Muscular Dystrophy (PPMD) and Duchenne UK for funding this work.”

The authors apologize for this error and state that this does not change the scientific conclusions of the article in any way. The original article has been updated.

