# Supplementary material for: Enhancing AAV-microdystrophin gene therapy after repeat dosing by blocking phagocytosis
Source: Front Immunol. 2025 Mar 3;16:1527840. doi: 10.3389/fimmu.2025.1527840 (PMC11911185; doi:10.3389/fimmu.2025.1527840)
Supplement: Supplementary file 1 [file DataSheet1.docx]

Supplementary Material

**Supplementary Figure 1. AAV9-microdystrophin viral genomes in the skeletal muscle are lower in mice receiving two rAAV doses.** DNA was purified from tibialis anterior muscle collected at euthanasia from mice receiving one (first dose) or two (second dose) AAV9-microdystrophin. 10 ng total DNA was subjected to Taqman qPCR, and the number of viral genomes was determined by generating a standard curve using titrated AAV9-µdys. Data are presented as mean ± SEM.

**Supplementary Figure 2. Anti-capsid antibody levels are higher in mice receiving two rAAV doses.** Sera (1:20,000) from terminal cardiac puncture from mice receiving one (first dose) or two (second dose) were applied in duplicate to 96-well Meso Scale Discovery (MSD) plates coated with 5 x 10^8 viral particles/well of AAV9 empty capsid (Charles River). MSD Sulfo-TAG labeled goat-anti mouse secondary antibody was added to each well and incubated for 1 hour. Bound signal (RFU) was detected on the MESO QuickPlex plate reader. Data are presented as mean ± SEM.

**Supplementary Figure 3. Anti-capsid antibodies after combination therapy.** Sera (1:20,000) from terminal cardiac puncture were applied in duplicate to 96-well Meso Scale Discovery (MSD) plates coated with 5 x 10^8 viral particles/well of AAV9 empty capsid (Charles River). MSD Sulfo-TAG labeled goat-anti mouse secondary antibody was added to each well and incubated for 1 hour. Bound signal (RFU) was detected on the MESO QuickPlex plate reader. Data are presented as mean ± SEM. AAV9-µDys, AAV9-microdystrophin; Ab, Antibody; includes complement receptor 1/2 antibody and CD11b antibody; TLR, Toll-like receptor.

**Supplementary Figure 4. Serum creatine kinase levels.** Sera from terminal cardiac puncture were assayed in duplicate using a commercially available CK kit (SEKISUI Diagnostics, LLC) according to the manufacturer’s instructions. Data are presented as mean ± SEM. AAV9-µDys, AAV9-microdystrophin; Ab, Antibody; includes complement receptor 1/2 antibody and CD11b antibody; TLR, Toll-like receptor.

**Supplementary Figure 5. Liver enzyme levels.** Sera from terminal cardiac puncture were assayed in duplicate using a commercially available ELISA kits (Abcam) according to manufacturer’s instructions. ALT, alanine amino transferase; AST, aspartate amino transferase; AAV9-µDys, AAV9-microdystrophin; Ab, Antibody; includes complement receptor 1/2 antibody and CD11b antibody; TLR, Toll-like receptor.

**Supplementary Table 1. Normalized tissue weight.**

|  | ***mdx*^a^** | ***mdx* + AAV9µdys^b^** | ***mdx* + AAV9µdys**  **+ αCR1/2/3 Abs**  **+TLR 7/8/9 antagonist^c^** | ***p*-value**  **(one way**  **ANOVA)** | **% change in tissue weight (b vs. c)** |
| --- | --- | --- | --- | --- | --- |
| **Spleen** | 0.336±0.008 | 0.296±0.015 | 0.316±0.010 | 0.08 | +6.9 |
| **Liver** | 1.073±0.037 | 1.096±0.069 | 1.166±0.079 | 0.59 | +6.4 |
| **Heart** | 0.507±0.005 | 0.484±0.017 | 0.491±0.014 | 0.51 | +1.4 |
| **Tricep** | 0.639±0.013 | 0.631±0.017 | 0.578±0.018 | 0.03* | -8.4% |
| **Quad** | 0.889±0.038 | 0.881±0.027 | 0.844±0.033 | 0.60 | -4.2% |
| **TA** | 0.217±0.010 | 0.216±0.006 | 0.207±0.003 | 0.54 | -4.2% |

**
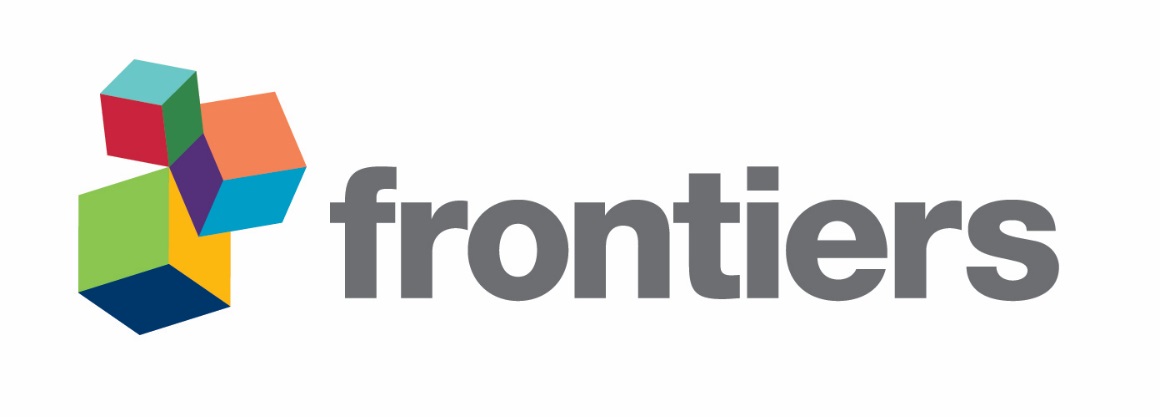
**
